# Parenting Under Pressure: How Child Limited Prosocial Emotions Shape the Stress-Warmth Connection

**DOI:** 10.1007/s10802-025-01338-6

**Published:** 2025-06-26

**Authors:** Nicholas D. Thomson, Sophie L. Kjaervik, Silvana Kaouar, Eva R. Kimonis

**Affiliations:** 1 Departments of Psychology, Psychiatry, and Surgery, Virginia Commonwealth University, 1200 E. Broad St, Richmond, VA USA; 2 Violence and Abuse, Norwegian Centre for Violence and Traumatic Stress Studies, Gullhaugveien 1-3, Oslo, 0484 Norway; 3 Parent-Child Research Clinic, School of Psychology, University of New South Wales, Sydney, Australia

**Keywords:** Parental warmth, Stress, Conduct disorder, Limited prosocial emotions

## Abstract

Parental warmth is a core intervention target for children with Limited Prosocial Emotions (LPE) whose conduct problems persist despite traditional psychosocial treatments. Research on factors uniquely influencing the capacity of parents of children with LPE to engage in warm parenting is limited but could advance treatment efforts for this persistently antisocial population. This study tested whether parents’ stress, a risk factor for disengaged, less nurturing, and sensitive parenting, uniquely influences parental warmth for youth with Conduct Disorder (CD) and LPE compared to CD alone. Participants included 126 youth (Age_Mean_ = 15.7 years, *SD* = 1.3, 70% boys, 55% African American/Black) diagnosed with CD and assessed for LPE via a multi-rater clinical interview. Youth rated maternal warmth at baseline and 6 months, caregivers self-reported general stress levels. Results of hierarchical linear regression analyses showed that LPE scores significantly moderated the association between mothers’ stress and warm parenting 6 months later, after controlling for initial warmth levels. Findings suggest that the robustly established link between parents’ stress and unresponsive, insensitive parenting is most pronounced for the subset of youth with CD + LPE. These findings can inform etiological models of LPE and point to potential treatment targets for improving outcomes for this aggressive youth population.

## Introduction

Parental warmth is a key treatment target of novel interventions aimed at improving outcomes for children with a severe form of Conduct Disorder (CD) characterized by Limited Prosocial Emotions (LPE; i.e., low empathy, remorse, uncaring attitudes, and shallow emotions collectively termed “callous-unemotional [CU] traits”). These children require nuanced intervention targeting different risk factors than their counterparts with CD and intact prosocial emotions because their Conduct Problem (CP) symptoms develop via distinct etiological pathways. The unique developmental trajectory of children with LPE explains why their CP symptoms tend to remain at clinically significant high levels after receiving treatments identified as efficacious for CDs. When these efficacious treatments, most notably Parent Management Training (PMT), are modified for children with CP + LPE they produce superior and sustained outcomes relative to standard PMT (Dadds et al., 2012). For example, children with CP + LPE treated with a PMT adaptation targeting their putative etiological factors, including parental warmth, sustained significant and large improvements in CP symptoms, whereas those receiving standard treatment deteriorated post-treatment.(Fleming et al., 2022) Despite superior outcomes relative to standard treatment, a substantial proportion of children continued showing clinically significant post-treatment CP levels. The current study investigates influences on parents’ ability to engage warmly with their children with LPE, focusing on parents’ stress, which offers the potential for refining targeted interventions and enhancing outcomes.

Parent stress is strongly implicated in the development and maintenance of child psychopathologies through its impact on parenting and parent-child relationship quality (Chiang & Bai, 2023; Neece et al., 2012). Higher stress levels among parents negatively influence parent-child interactions by lowering parents’ responsiveness, which predicts higher child CP and lower prosocial behavior (Conger et al., 2000; Ward & Lee, 2020). For example, parents who experienced more daily stressors were not only more likely to use negative parenting practices, but also less likely to engage in positive, warm, and responsive parenting behaviors with their children (Whiteside-Mansell et al., 2007). The family stress model (Conger et al., 2000, 2002) explains that stressors (e.g., financial, parenting-related) influence parents’ psychological states, affecting the quality of their interactions with their children and contributing to problematic child outcomes. More specifically, parental stress undermines effective parenting by reducing parents’ patience and coping abilities, making them more indifferent, reactive to, and punitive of negative child behaviors, and less engaged, nurturing, and sensitive to their child’s needs (Barreto et al., 2024; Elder et al., 1986; Landry et al., 2006).

As discussed above, low parental warmth is implicated as a key risk factor in the development of the LPE subtype of conduct disorders (Waller et al., 2018). Within diagnostic systems, LPE are a specifier to conduct disorders, including Oppositional Defiant Disorder (ODD) and Conduct Disorder (CD), with demonstrated clinical utility for signaling a more complex presentation involving early starting, severely aggressive, and persistent antisocial behavior that does not normalize with treatment (American Psychiatric Association, 2022; Perlstein et al., 2023; Viding & Kimonis, 2018; World health Organization, 2022). Whereas harsh and coercive parent-child interactions are central to the development of conduct disorders occurring when LPE are intact, findings are mixed about the relative importance of these dysfunctional parenting behaviors for children with LPE (Pasalich et al., 2011; Waller et al., 2018; Wootton et al., 1997). Instead, research more consistently converges on the importance of parental warmth in the development of callous antisociality (i.e., LPE; Pasalich et al., 2011; Wootton et al., 1997). For example, adolescents with LPE evoked fewer positive emotional expressions, interactions, and warmth from their parents than adolescents without LPE (Waller et al., 2014a). Furthermore, a large multi-site study found that while youth with CD + LPE consistently received lower parental warmth than those with CD only (Pauli et al., 2021). Thus, positive parenting behaviors distinguished the high CU group from the low CU group, suggesting that parents may struggle even more with providing warmth to youth who lack prosocial emotions than the challenges of dealing with conduct problems alone. Positive and warm parenting (e.g., encouragement, affection) is especially important for facilitating the development of prosocial attitudes, empathy, and conscience in children (Gardner et al., 2003; Kochanska, 1997), particularly for those with fearless temperaments who are at heightened risk for LPE (Colins et al., 2021; Goffin et al., 2018). Longitudinal studies find that when children with LPE experience lower levels of parental warmth, their levels of LPE worsen over time (Kroneman et al., 2011). Conversely, children with or at risk for LPE show lower levels of CP and improved LPE over time when exposed to warm parenting experiences (Waller et al., 2013). Despite the importance of parent warmth for moral socialization of temperamentally at-risk children, scant attention has been paid to factors influencing parents’ ability to engage in these critically important behaviors.

One such factor, parents’ stress, is found to be significantly greater for parents of children with LPE relative to their CP-only counterparts. For example, Enebrink et al. (2005) found that family stress was significantly higher among school-age children with LPE referred to psychiatric clinics than for those without LPE, even after controlling for their higher levels of aggressive behavior, ODD and/or CD symptoms. Similarly, mothers in a community sample of children with stable LPE reported significantly greater parental stress than children with moderate, decreasing, or low levels of LPE (Fanti & Centifanti, 2014). Furthermore, LPE moderated the relationship between parenting stress and child CP, such that they were only significantly associated in the LPE group but not the group with intact prosocial emotions. One explanation for this finding is that the combination of CP and a lack of concern from their child over their actions, is particularly distressing to parents and detrimental to the parent-child relationship.

The higher stress levels of parents of children with LPE may be a function of the problematic parent-child relationship. One study found that parent-child attachment difficulties contributed to higher parenting stress levels, which significantly predicted increased LPE (Fite et al., 2008). Such findings suggest that parents’ experience of stress may exacerbate already negative parent-child interactions, worsening warmth and responsiveness to positive child behavior, and thus interfering with moral socialization. Importantly, children with LPE exhibit behaviors such as emotional detachment, lack of remorse, and shallow affect, which can evoke particularly distressing cognitions in parents, including feeling emotionally rejected, ineffective, or even blamed for their child’s difficulties (Kaouar et al., 2024; Thomson, 2019). These parent perceptions may uniquely undermine warm engagement, especially under elevated stress when cognitive and emotional resources are strained. Consequently, the association between stress and reduced warmth may be more pronounced for parents of children with LPE, who may lack the typical social-emotional cues reinforcing parental efforts at warmth (Thomson et al., 2017). This theoretical rationale supports the hypothesis that child LPE may moderate the relation between parenting stress and parental warmth. Critically, much of the literature on parenting stress and LPE is cross-sectional and conducted on community samples who have low levels of CP and intact prosocial emotions, raising questions about the generalizability of these findings to more severe antisocial samples. Also, although longitudinal research supports that parenting stress predicts parental responsiveness (Coates & Phares, 2019; Ponnet et al., 2013; Ward & Lee, 2020), we were unable to locate any studies examining whether this relationship is moderated by LPE. Filling this knowledge gap is important for enhancing targeted interventions for complex CD presentations, such as by adjunctively intervening in parents’ stress. For example, anecdotal clinical experience with parents of children with CP + LPE engaged in treatment points to a negative impact of high-stress levels on parents’ ability to implement evidence-based relationship-building and behavioral management skills both in-clinic and during home practice with their child.

### The Current Study

In summary, parents’ stress influences parents’ ability to engage in warm and responsive parenting behaviors, and stress is greatest for parents of the subpopulation of children with CP + LPE who experience the lowest levels of parental warmth. Few studies have explored the role of moderators in the association between parenting stress and warm parenting behaviors, and more specifically, the influence of child LPE. Thus, the current study aimed to fill this knowledge gap by examining whether parents’ stress differentially predicts warm parenting behaviors for children with versus without LPE. Specifically, we examined whether LPE moderated the association between parents’ stress and maternal warmth six months later among a diverse, mixed-sex sample of adolescents diagnosed with CD. It was hypothesized that higher parents’ stress levels would significantly predict lower levels of later parental warmth, controlling for initial warmth levels, but only for youth with CD + LPE and not for youth with CD-only.

## Methods

### Participants

An *prior* power analysis using G*Power3 (Faul et al., 2007) for linear multiple regression, R^2^ increase, with power set to 0.95 and a medium effect size, indicated a required sample size of 107. We did not include 12 participants who declined to answer the questionnaires included. Thus, our final sample was 126 youth aged 13 to 17 (*M*_*age*_ = 15.70, *SD* = 1.32) diagnosed with conduct disorder and their maternal caregivers aged 29 to 67 (*M*_*age*_ = 44.69, *SD* = 8.37). Youth were primarily male (70%) and identified as African American/Black (52%), Caucasian/White (43%), both Black and White (3%), or American Indian or Alaska Native (2%).

### Procedure

Participants were recruited via a large healthcare network in Virginia. Prior to assessments, youth and caregivers were informed about the study and provided written assent and consent in separate and private rooms. This study is part of a larger project on how fear reactivity contributes to CU traits ([MASKED FOR REVIEW]). Assessments used in the present study were collected at baseline (time 1) and at 6 months post-baseline assessment (time 2). Participants were compensated $200 for their participation in the larger study. The study was approved by the Virginia Commonwealth University Institutional Review Board and received certification from the National Institutes of Health.

### Measures

#### Limited Prosocial Emotions

The Clinical Assessment of Prosocial Emotions (CAPE 1.1; Frick, 2013) is a clinical rating system for callous-unemotional symptoms that form the specifier “with Limited Prosocial Emotions” (LPE) in the criteria for Conduct Disorder in the 5th Edition of the Diagnostic and Statistical Manual of Mental Disorders (DSM-5; American Psychiatric Association, 2022). The CAPE 1.1. provides descriptions of the four CU symptoms: (1) lack of remorse or guilt, (2) callous lack of empathy, (3) unconcerned about performance, and (4) shallow or deficient affect. The LPE score was based on two semi-structured interviews – an informant (mother) and a self-report. The four symptoms are rated on a 3-point scale (0 = not at all descriptive, 2 = definitely descriptive). There are a series of follow-up questions that help determine symptoms. The number of symptoms scored as 2 is counted, and when more than two symptoms are scored as 2, the individual meets the criteria for LPE. The CAPE 1.1 has proven to be internally consistent across adolescents from clinic-referred and community samples, with alphas ranging from 0.73 to 0.82, and scores have been correlated with clinical diagnoses of oppositional defiant disorder and conduct disorder (Goetz et al., 2024; Hawes et al., 2020; Neo et al., 2023). In this study, 19% of youth met the criteria for LPE. All CAPE 1.1 assessments were conducted by clinical research coordinators who completed standardized training procedures in accordance with the CAPE manual (Frick, 2013), under the supervision of the first author (NDT). These procedures included didactic instruction on the conceptual and diagnostic basis of the Limited Prosocial Emotions (LPE) specifier, a detailed review of each of the four symptom domains (lack of remorse or guilt, callous lack of empathy, unconcerned about performance, and shallow or deficient affect), and training in the structure and scoring procedures of the CAPE. Clinical research staff participated in supervised practice sessions involving mock interviews and coding exercises using de-identified case vignettes, as well as weekly supervision and case review with NDT, who has both clinical and research experience administering the CAPE (e.g., Centifanti et al., 2019).

#### Maternal Warmth

The Quality of Parental Relationships Inventory (QPRI; Conger et al., 1994) is a youth-report assessment of parental relationships. In this study, we used the maternal warmth subscale, which included 9 items (e.g., “Acts loving and affectionate towards you” and “Listen carefully to your point of view”) rated on a 4-point scale (0 = *Never*, 3 = *Always*). The measure has proven to be internally consistent in samples of adults with an alpha of 0.80 (Backman et al., 2021), and the measure demonstrated excellent internal consistency in this study (a = 0.92). The inventory was administered at baseline and 6 months follow-up.

#### Parents Stress

The Perceived Stress Survey (PSS; Cohen, 1988) was completed by mothers to assess feelings and thoughts about stress during the last month (e.g., “In the last month, how often have you found that you could not cope with all the things that you had to do?” and “In the last month, how often have you felt difficulties were piling up so high that you could not overcome them?”). The scale includes 10 items rated on a 5-point scale (0 = never, 4 = very often). The scale has shown good reliability and validity in prior research with alphas ranging from 0.78 to 0.83, and scores have been correlated with other measures of stress (Baik et al., 2019). In this study, the scale demonstrated good internal consistency (a = 85).

#### Conduct Disorder

To covary with the model for CD symptoms, the Conduct Disorder subscale of the Proposed Specifiers of Conduct Disorder (PSCD; Salekin, 2016) was included. This is a self-report youth assessment of CD with 6 items rated on a 3-point scale (0 = Not true, 2 = True). The scale has proven to be internally consistent in samples of adolescents with alphas ranging from 0.70 to 0.77 and scores have been correlated with parent reports of conduct disorder (e.g., López-Romero et al., 2019; Muratori et al., 2021; Neumann et al., 2024;Salekin, 2022). Higher scores indicate increased levels of CD.

### Data Analysis Plan

A hierarchical regression was conducted to examine whether youths’ CU traits moderated the association between parents’ stress and maternal warmth 6 months later while controlling for youth age, sex, race, and conduct problems. Step 1 included all predictors and covariates. Step 2 included the interaction between youth LPE and parents’ stress. Analyses were performed using R studio (R Core Team, 2024). The simple slope was estimated using the ‘interactions’ package (Long, 2024), and the plot was created using the ‘sjPlot’ package (Lüdecke, 2024).

## Results

### Descriptive Statistics and Correlations

Correlations and descriptive statistics for study variables are displayed in Table 1. A positive correlation existed between maternal warmth at baseline and 6 months (*r* = .47, *p* <.001), suggesting stability over time. Maternal warmth at baseline was negatively related to LPE (*r* = −.24, *p* = .002) and youth age (*r* = −.19, *p* = .04), and positively related to youth sex (*r* = .18, *p* = .01), indicating children with CD only, who were younger, and boys perceived their maternal caregiver as warmer towards them. Maternal warmth at 6 months was negatively related to parents’ stress at baseline (*r* = −.25, *p* = .002), suggesting that parents’ stress reduced youth experience of maternal warmth.

**Table 1:** Correlations and descriptive statistics

	1	2	3	4	5	6	7	8	9
1. Age (caregiver)	-								
2. Age (youth)	0.05	-							
3. Sex^a^	− 0.006	− 0.15	-						
4. Race^a^	− 0.19*	0.13	− 0.22**	-					
5. CD	− 0.04	0.14	0.06	− 0.08	-				
6. LPE^a^	− 0.03	0.04	0.06	− 0.05	0.18*	-			
7. Parents’ stress	− 0.21*	0.03	− 0.15	0.11	0.17	0.10	-		
8. Warmth (T1)	− 0.08	− 0.19*	0.21*	− 0.06	− 0.20*	− 0.20**	− 0.15	-	
9. Warmth (T2)	0.02	0.001	0.08	− 0.07	− 0.17	− 0.08	− 0.17	0.44***	-
*Mean*	44.69	15.70	0.70	0.55	3.53	0.19	17.21	20.13	20.32
*SD*	8.37	1.32	0.46	0.50	2.92	0.39	6.91	5.81	7.13
Skewness	0.63	-0.55	-0.85	-0.19	0.86	1.56	-0.27	-0.72	-0.87
Kurtosis	-0.33	-0.96	-1.28	-1.98	-0.09	0.43	-0.73	-0.06	-0.39

Note. Sex = (0) female, (1) male. Race = (0) other, (1) African-American. T1 = Baseline; T2 = Follow-up at 6 months. categorical variables^a^.*p <.05, **p <.01, ***p <.001.

### Parent Stress, Maternal Warmth, and LPE

**Fig. 1:**
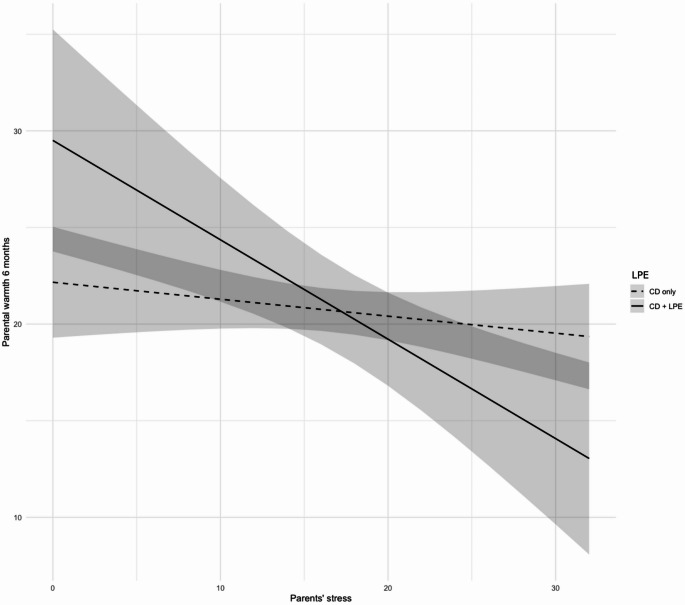
The interaction between LPE and parents’ stress predicts maternal warmth T2

The regression results are displayed in Table 2. Step 1 was significant, *F*(7, 116) = 7.48, *p* <.001, and lower levels of parents’ stress (*p* = .02) and higher levels of time 1 maternal warmth (*p* <.001) predicted higher time 2 maternal warmth. Step 2, which included the interaction term between parents’ stress and youth LPE, was also significant, *F*(8, 115) = 7.32, *p* <.001, and the interaction term was significant (*p* = .035). Figure 1 displays the simple slope analysis, which revealed that the negative effect of parents’ stress on warmth was significant for parents of youth with CD + LPE, *b* = -0.52, *SE* = 0.17, *p* <.001, but not for parents of youth with CD only, *b* = -0.10, *SE* = 0.09, *p* = .27. Thus, parents of children with CD + LPE who experience higher levels of stress tended to display significantly lower maternal warmth at time 2 (controlling for baseline warmth), compared to parents of children with CD only.

**Table 2:** Regressions for LPE and parents’ stress predicting maternal warmth 6 months later

	b	SE	b	*R* ^2^
**Step 1**				0.27***
Age	0.11	0.43	0.02	
Sex	-0.67	1.25	-0.04	
Race	0.31	1.12	0.02	
CD	-0.16	0.19	-0.07	
Warmth (T1)	0.64	0.11	0.51***	
LPE	1.00	1.41	0.06	
Parents stress	-0.19	0.08	-0.19*	
**Step 2**				0.29***
Age	0.23	0.43	0.04	
Sex	-0.61	1.23	-0.04	
Race	0.20	1.11	0.01	
CD	-0.11	0.19	-0.04	
Warmth (T1)	0.60	0.10	0.48***	
LPE	8.32	3.69	0.48*	
Parent stress	-0.10	0.09	-0.10	
LPE*Parents stress	-0.41	0.19	-0.48*	

Note. *p <.05, **p <.01, ***p <.001.

## Discussion

Little is known about how parents’ stress influences parents’ capacity to engage in warm parenting behaviors for the subset of youth with LPE who robustly show significantly poorer levels of both constructs. The purpose of the current study was to address this knowledge gap by examining whether LPE moderates the relationship between parents’ stress and parental warmth six months later among children with CD. Results indicated that higher levels of maternal caregivers stress predicted lower future levels of youth perceived maternal warmth, but only for those diagnosed with CD + LPE and not for youth with CD-alone. Study strengths included multi-rater and multi-method assessment using tools with strong psychometric properties within a mixed-gender sample of adolescents diagnosed with CD, increasing the generalizability of findings to other antisocial and clinical populations.

Our findings suggest that the negative impact of parents’ stress on parenting behaviors is most relevant to the subset of children with CD who meet the diagnostic criteria for the LPE specifier. These findings shed light on prior studies reporting that parents’ stress influences parental responsiveness by clarifying that this link may not apply to all youth but rather specifically to those with poorly developed prosocial tendencies. The consistency of this finding that parents’ stress influences warmth in our adolescent sample, as was previously found in low-income samples of young children (Dau et al., 2019; Ward & Lee, 2020), speaks to the robustness of this effect. It also highlights the continuing impact of parents’ stress on parenting behaviors across development, from early childhood into adolescence, potentially offering multiple opportunities for intervention. In addition to the numerous demands and challenges that parents of typically developing children experience day-to-day, those of children with CD + LPE must respond to extreme, serious, and escalating behavior that not only appears unchanged by typical forms of punishment (Kimonis et al., 2024) but can cause considerable harm to others for which the youth shows little care, concern, or reparative behavior (Pardini, 2011). Furthermore, due to the damage and isolation caused to the family’s social networks by these behaviors, these caregivers may have even fewer available resources and support to cope with their exceptionally high parenting demands (Abidin, 1992). Where parents struggle with their own mental health issues, resources may be even further depleted.

Our findings set the groundwork for further interrogation of the role of parents’ stress in the developmental psychopathology of LPE, which is likely to be complex. For example, a recent systematic review of 29 mostly cross-sectional studies identified many factors influencing parents’ stress levels, with the strongest effects identified for parents’ psychopathology and personality, child’s difficult temperament, and conduct problems (Fang et al., 2024). Furthermore, parents’ stress directly and indirectly influences parents’ engagement in positive parenting behaviors via various mediating variables (e.g., self-regulation skills, such as psychological flexibility within parenting; Fonseca et al., 2020). Caregiver emotional and behavioral regulation in the face of stressors is critical to promoting the optimal warm but firm, authoritative parenting style (Baumrind, 1991) and buffering the direct and contagion effects of toxic stressors on the child’s developing brain (Gunnar & Hostinar, 2015). However, these relationships have yet to be examined in the context of stress experienced by parents who are caregivers of children with LPE. Our findings encourage further research to better understand these complex relationships and unravel exactly how the various implicated factors contribute to child antisocial behavior that co-occurs with poor prosocial development.

### Theoretical and Practical Implications

Theoretically, our findings highlight the importance of considering how parent-specific stressors contribute to the poor parent-child relationships of children with CU traits, impacting parents’ ability to express warmth and be responsive to their child’s needs. To date, more emphasis has been placed in the literature on these parenting behaviors themselves than on third factors that might influence them (Pasalich et al., 2011; Waller et al., 2013). Some parents’ capacity to engage in warm and responsive parenting may be limited if stressors are not first addressed. Practically, parent-focused treatment programs for CP + LPE should consider integrating components focused on alleviating parents’ stress. For example, Parent-Child Interaction Therapy (PCIT), adapted for children with CP + LPE (called ‘PCIT-CU’), incorporates a focus on parents’ stress from its third parent-child coaching session. In this treatment session, parents are provided psychoeducation on how stress impacts the capacity of parents to effectively apply evidence-based relationship-building and behavior-management strategies taught didactically and directly applied using in-vivo bug-in-ear coaching. Parents are encouraged to generate and practice during the week small, achievable stress relief strategies from a parent “menu,” which therapists follow up on and reinforce parents’ use of in future PCIT-CU sessions. However, implementing this stress relief component is likely variable across treating clinicians and dependent on the time available for these parent discussions during brief check-in and debrief sessions around intensive in-vivo parent coaching. That is, parents of children who are more disruptive, which is more characteristic of those with LPE, are likely to have less time available to discuss and problem-solve their use of daily stress relief strategies with the therapist. Consequently, targeted interventions like PCIT-CU could be enhanced by incorporating a more systematic parent stress relief intervention.

The parents’ stress component of PCIT-CU was built on limited research at the time of the protocol’s development, which reported higher stress levels among parents of children with elevated CU traits. However, the body of research has since grown to provide a more nuanced understanding of what aspects of parents’ stress relate to LPE. For example, among a clinic-referred sample of children with conduct problems, Kaouar et al. (2024) found that parents’ stress related to a dysfunctional parent-child relationship (i.e., distanced, not meeting parents’ expectations or perceived as reinforcing to the parent) was specifically associated with secondary LPE (with comorbid anxiety), and to a greater extent than for children with CP-only or primary LPE (presenting in the absence of comorbid anxiety). Other contemporary research from the parenting and LPE field highlights the greater maladaptive cognitions of parents of children with LPE, which may further influence the link between parents’ stress and the quality of parent-child interactions and parenting behaviors. For example, Kaouar et al. (2024) found that parents of children with LPE held less warm cognitions about their children than those with children with CP-only. Understanding whether parents’ cognitions characterized by low warmth underpin less warm parenting behaviors could aid clinicians in providing parents with beneficial psychoeducation and potentially directly targeting these cognitions within treatments such as PCIT-CU (Fleming et al., 2022). Such longitudinal intervention research can directly test the mechanistic role of parents’ stress on parental warmth, an avenue for future research.

While this study focused on parental warmth as the parenting behavior impacted by stress, other parenting constructs, such as monitoring, involvement, and conflict, are also important during adolescence (Stattin & Kerr, 2000). The focus on warmth was driven by research showing that children with CU traits, particularly those with diagnosed LPE, are less responsive to behavioral control strategies and more sensitive to emotional tone and connection (Waller et al., 2013; Frick & Morris, 2004). Nonetheless, the absence of other parenting outcomes limits the ability to determine whether the observed moderation is specific to warmth, or whether parents stress similarly undermine other key parenting behaviors. Future research should include other parenting behaviors to better understand how parents stress differentially influences the parent-child relationship across domains in adolescence.

Another important consideration is that the relationship between parents stress, child LPE, and parental warmth may be reciprocal and transactional (Sameroff, 2009). Rather than a one-way street, each factor may feed back into the other, creating a self-perpetuating cycle. Indeed, some longitudinal research indicates a bidirectional effect, where higher parental warmth at age 2 predicted lower CU traits at age 3, and conversely, toddlers with higher CU traits evoked less maternal warmth one year later (Waller et al., 2014b). Although this study was conducted with toddlers, this cycle likely continues into adolescence and may, over time, become increasingly difficult to break, especially if external stress is continually high. Thus, Waller et al. (2014b) concluded that these behaviors show some malleability, and that parenting adapts to child behavior. Thus, if either the maternal caregiver learns to better tackle stress and use warm parenting, or the child can learn to show empathy, the other might respond in kind, gradually improving the cycle.

### Limitations

Our findings must be considered within the context of some study limitations. First, while yielding significant power to detect our interaction effect, the relatively small sample size was not adequately powered to examine primary and secondary variants of LPE (Kimonis, 2023). Second, we only assessed maternal caregivers’ stress levels. It will be important for future research to also examine these relationships among fathers, given some nuanced influences on CP outcomes, but generally, similar findings that their stress levels impact child prosocial and cognitive outcomes via influencing the quality of parent-child interactions and parenting behaviors (Ward & Lee, 2020). Traditionally, mothers also spend more time caring for children relative to fathers, pointing to possibly having a greater impact on their outcomes, although possibly more so in earlier childhood (Jones & Mosher, 2013). Future research with larger samples may also examine how child sex and parent sex interact in predicting these associations, given prior research findings that parents of girls experience less parenting stress in early childhood relative to parents of boys (Williford et al., 2007). Finally, we did not have a measure of parents’ psychopathology to include as a covariate in analyses, given prior findings of its association with parents’ stress (Aviles et al., 2024; Fang et al., 2024), necessitating further research.

## Conclusion

Stress affects a large proportion of parents (Raphael et al., 2010), particularly those from low-income households (Neppl et al., 2016) and those with children with CD + LPE (Fanti & Centifanti, 2014). High stress levels undermine positive parenting behaviors by influencing the quality of parent-child interactions, further impacting prosocial development. With the emergence of targeted interventions designed to address non-response among children with complex CD that present with LPE (Fleming et al., 2022; Perlstein et al., 2023), it is important to identify those factors affecting parents’ ability to engage in the therapeutic process. For example, some research finds that parents of children with LPE are more likely to drop out of treatment than children without LPE (Högström et al., 2013). Our findings forge the way for future research investigating factors that influence treatment engagement and outcomes for families of children with LPE.

## References

[CR1] Abidin, R. R. (1992). The determinants of parenting behavior. *Journal of Clinical Child Psychology*, *21*(4), 407–412. 10.1207/s15374424jccp2104_12

[CR2] *Diagnostic and Statistical Manual of Mental Disorders | Psychiatry Online*. DSM American Psychiatric Association, & Library (2022). https://psychiatryonline.org/doi/book/10.1176/appi.books.9780890425787

[CR3] Aviles, A. I., Betar, S. K., Cline, S. M., Tian, Z., Jacobvitz, D. B., & Nicholson, J. S. (2024). Parenting young children during COVID-19: Parenting stress trajectories, parental mental health, and child problem behaviors. *Journal of Family Psychology*, *38*(2), 296–308. 10.1037/fam0001181 38236275 10.1037/fam0001181

[CR4] Backman, H., Laajasalo, T., Jokela, M., & Aronen, E. T. (2021). Parental warmth and hostility and the development of psychopathic behaviors: A longitudinal study of young offenders. *Journal of Child and Family Studies*, *30*(4), 955–965. 10.1007/s10826-021-01921-7

[CR5] Baik, S. H., Fox, R. S., Mills, S. D., Roesch, S. C., Sadler, G. R., Klonoff, E. A., & Malcarne, V. L. (2019). Reliability and validity of the perceived stress Scale-10 in Hispanic Americans with english or Spanish Language preference. *Journal of Health Psychology*, *24*(5), 628–639. 10.1177/1359105316684938 28810432 10.1177/1359105316684938 PMC6261792

[CR6] Barreto, S., Wang, S., Guarnaccia, U., Fogelman, N., Sinha, R., & Chaplin, T. M. (2024). Parent stress and observed parenting in a Parent-Child interaction task in a predominantly minority and Low-Income sample. *Archives of Pediatrics (Lisle IL)*, *9*(1), 308. 10.29011/2575-825x.100308 38939555 10.29011/2575-825x.100308 PMC11209751

[CR7] Baumrind, D. (1991). The influence of parenting style on adolescent competence and substance use. *The Journal of Early Adolescence*, *11*(1), 56–95. 10.1177/0272431691111004

[CR9] Centifanti, L. C. M., Shaw, H., Atherton, K. J., Thomson, N. D., MacLellan, S., & Frick, P. J. (2019). CAPE for measuring callous-unemotional traits in disadvantaged families: A cross-sectional validation study. *F1000Research*, *8*, 1027. 10.12688/f1000research.19605.1 10.12688/f1000research.19605.1 PMC7059787 32185018

[CR10] Chiang, S. C., & Bai, S. (2023). Bidirectional associations between parenting stress and child psychopathology: The moderating role of maternal affection. *Development and Psychopathology*, 1–11. 10.1017/S0954579423001177 10.1017/S0954579423001177 PMC10978553 37771133

[CR11] Coates, E. E., & Phares, V. (2019). Pathways linking nonresident father involvement and child outcomes. *Journal of Child and Family Studies*, *28*(6), 1681–1694. 10.1007/s10826-019-01389-6

[CR12] Cohen, S. (1988). Perceived stress in a probability sample of the united States. *The social psychology of health* (pp. 31–67). Sage Publications, Inc.

[CR13] Colins, O. F., Fanti, K. A., & Andershed, H. (2021). The DSM-5 limited prosocial emotions specifier for conduct disorder: Comorbid problems, prognosis, and antecedents. *Journal of the American Academy of Child & Adolescent Psychiatry*, *60*(8), 1020–1029. 10.1016/j.jaac.2020.09.022 33068752 10.1016/j.jaac.2020.09.022

[CR15] Conger, R. D., Ge, X., Elder, G. H., Lorenz, F. O., & Simons, R. L. (1994). Economic stress, coercive family process, and developmental problems of adolescents. *Child Development*, *65*(2 Spec No). https://pubmed.ncbi.nlm.nih.gov/8013239/8013239

[CR14] Conger, K. J., Rueter, M. A., & Conger, R. D. (2000). The role of economic pressure in the lives of parents and their adolescents: The family stress model. In L. J. Crockett, & R. K. Silberiesen (Eds.), *Negotiating adolescence in times of social change* (pp. 201–223). Cambridge University Press.

[CR16] Conger, R. D., Wallace, L. E., Sun, Y., Simons, R. L., McLoyd, V. C., & Brody, G. H. (2002). Economic pressure in African American families: A replication and extension of the family stress model. *Developmental Psychology*, *38*(2), 179–193. 10.1037/0012-1649.38.2.179 11881755

[CR17] Dadds, M. R., Cauchi, J. C., Wimalaweera, S., Hawes, D. J., & Brennan, J. (2012). Outcomes, moderators, and mediators of empathic-emotion recognition training for complex conduct problems in childhood. *Psychiatry Research*, *199*(3). 10.1016/j.psychres.2012.04.033 10.1016/j.psychres.2012.04.033 22703720

[CR8] Dau, B. T., Callinan, A. L. B. T., L. S., & Smith, M. V. (2019). An examination of the impact of maternal fetal attachment, postpartum depressive symptoms and parenting stress on maternal sensitivity. *Infant Behavior and Development*, *54*, 99–107. 10.1016/j.infbeh.2019.01.001 30658270 10.1016/j.infbeh.2019.01.001

[CR18] Elder, G. H., Caspi, A., & van Nguyen, T. (1986). Resourceful and Vulnerable Children: Family Influence in Hard Times. In R. K. Silbereisen, K. Eyferth, & G. Rudinger (Eds.), *Development as Action in Context: Problem Behavior and Normal Youth Development* (pp. 167–186). Springer. 10.1007/978-3-662-02475-1_9

[CR19] Enebrink, P., Andershed, H., & Långström, N. (2005). Callous-unemotional traits are associated with clinical severity in referred boys with conduct problems. *Nordic Journal of Psychiatry*, *59*(6), 431–440. 10.1080/08039480500360690 16316895 10.1080/08039480500360690

[CR20] Fang, Y., Luo, J., Boele, M., Windhorst, D., van Grieken, A., & Raat, H. (2024). Parent, child, and situational factors associated with parenting stress: A systematic review. *European Child & Adolescent Psychiatry*, *33*(6), 1687–1705. 10.1007/s00787-022-02027-1 35876894 10.1007/s00787-022-02027-1 PMC11211171

[CR21] Fanti, K. A., & Centifanti, L. C. M. (2014). Childhood callous-unemotional traits moderate the relation between parenting distress and conduct problems over time. *Child Psychiatry and Human Development*, *45*(2), 173–184. 10.1007/s10578-013-0389-3 25879085 10.1007/s10578-013-0389-3

[CR22] Fite, P. J., Greening, L., & Stoppelbein, L. (2008). Relation between parenting stress and psychopathic traits among children. *Behavioral Sciences & the Law*, *26*(2), 239–248. 10.1002/bsl.803 18344170 10.1002/bsl.803

[CR23] Fleming, G. E., Neo, B., Briggs, N. E., Kaouar, S., Frick, P. J., & Kimonis, E. R. (2022). Parent training adapted to the needs of children with Callous-Unemotional traits: A randomized controlled trial. *Behavior Therapy*, *53*(6), 1265–1281. 10.1016/j.beth.2022.07.001 36229121 10.1016/j.beth.2022.07.001

[CR24] Fonseca, A., Moreira, H., & Canavarro, M. C. (2020). Uncovering the links between parenting stress and parenting styles: The role of psychological flexibility within parenting and global psychological flexibility. *Journal of Contextual Behavioral Science*, *18*, 59–67. 10.1016/j.jcbs.2020.08.004

[CR25] Frick, P. J. (2013). *Clinical Assessment of Prosocial Emotions: Version 1.1(CAPE 1.1)* [{Measurement instrument}]. https://sites01.lsu.edu/faculty/pfricklab/wp-content/uploads/sites/100/2015/11/CAPE-Manual.pdf

[CR26] Gardner, F., Ward, S., Burton, J., & Wilson, C. (2003). The role of mother-child joint play in the early development of children’s conduct problems: A longitudinal observational study. *Social Development*, *12*(3), 361–378. 10.1111/1467-9507.00238

[CR27] Goetz, C. M., Miller, T. A., & Frick, P. J. (2024). The clinical assessment of prosocial emotions (CAPE): Initial tests of reliability and validity in a clinic-referred sample of children and adolescents. *Psychological Assessment*, *36*(8), 452–461. 10.1037/pas0001320 38709629 10.1037/pas0001320

[CR28] Goffin, K. C., Boldt, L. J., Kim, S., & Kochanska, G. (2018). A unique path to Callous-Unemotional traits for children who are temperamentally fearless and unconcerned about transgressions: A longitudinal study of typically developing children from age 2 to 12. *Journal of Abnormal Child Psychology*, *46*(4), 769–780. 10.1007/s10802-017-0317-2 28608168 10.1007/s10802-017-0317-2 PMC5729059

[CR29] Gunnar, M. R., & Hostinar, C. E. (2015). The social buffering of the hypothalamic-pituitary-adrenocortical axis in humans: Developmental and experiential determinants. *Social Neuroscience*, *10*(5), 479–488. 10.1080/17470919.2015.1070747 26230646 10.1080/17470919.2015.1070747 PMC4618716

[CR30] Hawes, D. J., Kimonis, E. R., Diaz, M., Frick, A., P. J., & Dadds, M. R. (2020). The clinical assessment of prosocial emotions (CAPE 1.1): A multi-informant validation study. *Psychological Assessment*, *32*(4), 348–357. 10.1037/pas0000792 31829639 10.1037/pas0000792

[CR31] Högström, J., Enebrink, P., & Ghaderi, A. (2013). The moderating role of child callous-unemotional traits in an Internet-based parent-management training program. *Journal of Family Psychology: JFP: Journal of the Division of Family Psychology of the American Psychological Association (Division 43)*, *27*(2), 314–323. 10.1037/a0031883 23458700 10.1037/a0031883

[CR32] Jones, J., & Mosher, W. D. (2013). Fathers’ involvement with their children: United states, 2006–2010. *National Health Statistics Reports*, *71*, 1–21. 24467852

[CR33] Kaouar, S., Fleming, G. E., Neo, B., Hawes, D. J., Eapen, V., & Kimonis, E. R. (2024). Dimensions of warm parenting attributions differentiate conduct problem subtypes in young children. *Research on Child and Adolescent Psychopathology*, *52*(2), 223–236. 10.1007/s10802-023-01111-7 37581855 10.1007/s10802-023-01111-7 PMC10834570

[CR34] Kimonis, E. R. (2023). The emotionally sensitive child-adverse parenting experiences-allostatic (over)load (ESCAPE-AL) model for the development of secondary psychopathic traits. *Clinical Child and Family Psychology Review*, *26*(4), 1097–1114. 10.1007/s10567-023-00455-2 37735279 10.1007/s10567-023-00455-2 PMC10640461

[CR35] Kimonis, E. R., Cooper, F., Neo, B., Fleming, G. E., Chan, M. E., McDonogh, C., & Bressel, J. R. D., P (2024). Affective and behavioral responses to time out in preschool children with conduct problems and varying levels of Callous-Unemotional traits. *Behavior Therapy*. 10.1016/j.beth.2024.07.005 40010910 10.1016/j.beth.2024.07.005

[CR36] Kochanska, G. (1997). Multiple pathways to conscience for children with different temperaments: From toddlerhood to age 5. *Developmental Psychology*, *33*(2), 228–240. 10.1037/0012-1649.33.2.228 9147832 10.1037//0012-1649.33.2.228

[CR37] Kroneman, L. M., Hipwell, A. E., Loeber, R., Koot, H. M., & Pardini, D. A. (2011). Contextual risk factors as predictors of disruptive behavior disorder trajectories in girls: The moderating effect of Callous-Unemotional features. *Journal of Child Psychology and Psychiatry and Allied Disciplines*, *52*(2), 167–175. 10.1111/j.1469-7610.2010.02300.x 20735513 10.1111/j.1469-7610.2010.02300.x PMC2994952

[CR38] Landry, S. H., Smith, K. E., & Swank, P. R. (2006). Responsive parenting: Establishing early foundations for social, communication, and independent problem-solving skills. *Developmental Psychology*, *42*(4), 627–642. 10.1037/0012-1649.42.4.627 16802896 10.1037/0012-1649.42.4.627

[CR39] Long, J. A. (2024). *interactions: Comprehensive, User-Friendly Toolkit for Probing Interactions* (Version 1.2.0) [Computer software]. https://cran.r-project.org/web/packages/interactions/index.html

[CR40] López-Romero, L., Romero, E., Colins, O. F., Andershed, H., Hare, R. D., & Salekin, R. T. (2019). Proposed specifiers for conduct disorder (PSCD): Preliminary validation of the parent version in a Spanish sample of preschoolers. *Psychological Assessment*, *31*(11), 1357–1367. 10.1037/pas0000759 31368737 10.1037/pas0000759

[CR41] Muratori, P., Buonanno, C., Gallani, A., Grossi, G., Levantini, V., Milone, A., Pisano, S., Salekin, R. T., Sesso, G., Masi, G., & Nocentini, A. (2021). Validation of the proposed specifiers for conduct disorder (PSCD) scale in a sample of Italian students. *Children (Basel Switzerland)*, *8*(11), 1020. 10.3390/children8111020 34828733 10.3390/children8111020 PMC8622648

[CR42] Neece, C. L., Green, S. A., & Baker, B. L. (2012). Parenting stress and child behavior problems: A transactional relationship across time. *American Journal on Intellectual and Developmental Disabilities*, *117*(1), 48–66. 10.1352/1944-7558-117.1.48 22264112 10.1352/1944-7558-117.1.48 PMC4861150

[CR43] Neo, B., Fleming, G. E., Kaouar, S., Chan, M. E., Huang, N. N., Hawes, D. J., Eapen, V., Briggs, N., & Kimonis, E. R. (2023). Clinical utility of diagnosing limited prosocial emotions in young children using the clinical assessment of prosocial emotions (CAPE). *Psychological Assessment*, *35*(12), 1085–1097. 10.1037/pas0001279 37768639 10.1037/pas0001279

[CR44] Neppl, T. K., Senia, J. M., & Donnellan, M. B. (2016). Effects of economic hardship: Testing the family stress model over time. *Journal of Family Psychology: JFP: Journal of the Division of Family Psychology of the American Psychological Association (Division 43)*, *30*(1), 12–21. 10.1037/fam0000168 26551658 10.1037/fam0000168 PMC4742411

[CR45] Neumann, C. S., Salekin, R. T., Commerce, E., Charles, N. E., Barry, C. T., Mendez, B., & Hare, R. D. (2024). Proposed specifiers for conduct disorder (PSCD) scale: A latent profile analysis with At-Risk adolescents. *Research on Child and Adolescent Psychopathology*, *52*(3), 369–383. 10.1007/s10802-023-01126-0 37922002 10.1007/s10802-023-01126-0

[CR46] Pardini, D. (2011). Perceptions of social conflicts among incarcerated adolescents with callous-unemotional traits: You’re going to pay. It’s going to hurt, but I don’t care. *Journal of Child Psychology and Psychiatry*, *52*(3), 248–255. 10.1111/j.1469-7610.2010.02336.x 21073459 10.1111/j.1469-7610.2010.02336.x PMC3034798

[CR47] Pasalich, D. S., Dadds, M. R., Hawes, D. J., & Brennan, J. (2011). Do callous-unemotional traits moderate the relative importance of parental coercion versus warmth in child conduct problems? An observational study. *Journal of Child Psychology and Psychiatry*, *52*(12), 1308–1315. 10.1111/j.1469-7610.2011.02435.x 21726225 10.1111/j.1469-7610.2011.02435.x

[CR48] Pauli, R., Tino, P., Rogers, J. C., Baker, R., Clanton, R., Birch, P., Brown, A., Daniel, G., Ferreira, L., Grisley, L., Kohls, G., Baumann, S., Bernhard, A., Martinelli, A., Ackermann, K., Lazaratou, H., Tsiakoulia, F., Bali, P., Oldenhof, H., & De Brito, S. A. (2021). Positive and negative parenting in conduct disorder with high versus low levels of callous-unemotional traits. *Development and Psychopathology*, *33*(3), 980–991. 10.1017/S0954579420000279 32571444 10.1017/S0954579420000279

[CR49] Perlstein, S., Fair, M., Hong, E., & Waller, R. (2023). *Treatment of childhood disruptive behavior disorders and callous-unemotional traits: A systematic review and two multilevel meta‐analyses*. *64*(9), 1372–1387. 10.1111/jcpp.13774 PMC10471785 36859562

[CR50] Ponnet, K., Mortelmans, D., Wouters, E., Van Leeuwen, K., Bastaits, K., & Pasteels, I. (2013). Parenting stress and marital relationship as determinants of mothers’ and fathers’ parenting. *Personal Relationships*, *20*(2), 259–276. 10.1111/j.1475-6811.2012.01404.x

[CR51] R Core Team (2024). *R: A language and environment for statistical computing. R Foundation for Statistical Computing, Vienna, Austria.* https://www.R-project.org/

[CR52] Raphael, J. L., Zhang, Y., Liu, H., & Giardino, A. P. (2010). Parenting stress in US families: Implications for paediatric healthcare utilization. *Child: Care Health and Development*, *36*(2), 216–224. 10.1111/j.1365-2214.2009.01052.x 20047600 10.1111/j.1365-2214.2009.01052.x

[CR53] Salekin, R. T. (2016). Psychopathy in childhood: Toward better informing the DSM–5 and ICD-11 conduct disorder specifiers. *Personality Disorders: Theory Research and Treatment*, *7*(2), 180–191. 10.1037/per0000150 10.1037/per0000150 26389622

[CR54] Salekin, R. T. (2022). Some critical comments on the Frick (2022) paper titled some critical considerations in applying the construct of psychopathy to research and classification of childhood disruptive behavior disorders. *Clinical Psychology Review*, *98*, 102214. 10.1016/j.cpr.2022.102214 36328895 10.1016/j.cpr.2022.102214

[CR55] Sameroff, A. (2009). The transactional model. In *The transactional model of development: How children and contexts shape each other* (pp. 3–21). American Psychological Association. 10.1037/11877-001

[CR56] Thomson, N. D. (2019). *Understanding psychopathy: The biopsychosocial perspective*. Routledge.

[CR57] Thomson, N. D., Centifanti, L. C. M., & Lemerise, E. A. (2017). Emotion regulation and conduct disorder: The role of callous-unemotional traits. *Emotion regulation and psychopathology in children and adolescents* (pp. 129–153). Oxford University Press. 10.1093/med:psych/9780198765844.003.0007

[CR58] Viding, E., & Kimonis, E. R. (2018). Callous–unemotional traits. *Handbook of psychopathy* (2nd ed., pp. 144–164). The Guilford Press.

[CR59] Waller, R., Gardner, F., & Hyde, L. W. (2013). What are the associations between parenting, callous-unemotional traits, and antisocial behavior in youth? A systematic review of evidence. *Clinical Psychology Review*, *33*(4). 10.1016/j.cpr.2013.03.001 10.1016/j.cpr.2013.03.001 23583974

[CR60] Waller, R., Gardner, F., Viding, E., Shaw, D. S., Dishion, T. J., Wilson, M. N., & Hyde, L. W. (2014a). Bidirectional associations between parental warmth, callous unemotional behavior, and behavior problems in High-Risk preschoolers. *Journal of Abnormal Child Psychology*, *42*(8), 1275–1285. 10.1007/s10802-014-9871-z 24740437 10.1007/s10802-014-9871-z PMC4804198

[CR61] Waller, R., Gardner, F., Viding, E., Shaw, D. S., Dishion, T. J., Wilson, M. N., & Hyde, L. W. (2014b). Bidirectional associations between parental warmth, callous unemotional behavior, and behavior problems in high-risk preschoolers. *Journal of Abnormal Child Psychology*, *42*(8), 1275–1285. 10.1007/s10802-014-9871-z 24740437 10.1007/s10802-014-9871-z PMC4804198

[CR62] Waller, R., Hyde, L. W., Klump, K. L., & Burt, S. A. (2018). Parenting is an environmental predictor of Callous-Unemotional traits and aggression: A monozygotic twin differences study. *Journal of the American Academy of Child & Adolescent Psychiatry*, *57*(12), 955–963. 10.1016/j.jaac.2018.07.882 30522741 10.1016/j.jaac.2018.07.882 PMC6296820

[CR63] Ward, K. P., & Lee, S. J. (2020). Mothers’ and fathers’ parenting stress, responsiveness, and child wellbeing among Low-Income families. *Children and Youth Services Review*, *116*. 10.1016/j.childyouth.2020.105218 10.1016/j.childyouth.2020.105218 PMC7425837 32801410

[CR64] Whiteside-Mansell, L., Ayoub, C., McKelvey, L., Faldowski, R. A., Hart, A., & Shears, J. (2007). Parenting stress of low-income parents of toddlers and preschoolers: Psychometric properties of a short form of the parenting stress index. *Parenting: Science and Practice*, *7*(1), 27–56. 10.1207/s15327922par0701_2

[CR65] Williford, A. P., Calkins, S. D., & Keane, S. P. (2007). Predicting change in parenting stress across early childhood: Child and maternal factors. *Journal of Abnormal Child Psychology*, *35*(2), 251–263. 10.1007/s10802-006-9082-3 17186365 10.1007/s10802-006-9082-3

[CR66] Wootton, J. M., Frick, P. J., Shelton, K. K., & Silverthorn, P. (1997). Ineffective parenting and childhood conduct problems: The moderating role of callous-unemotional traits. *Journal of Consulting and Clinical Psychology*, *65*(2), 301–308. 10.1037/0022-006X.65.2.292.b 9086694 10.1037/0022-006x.65.2.292.b

[CR67] World health Organization (2022). *ICD-11: International classification of diseases (11th revision)*. https://icd.who.int/en

[CR68] Faul, F., Erdfelder, E., Lang, A. G., & Buchner, A. (2007). G* Power 3: A flexible statistical power analysis program for the social, behavioral, and biomedical sciences. *Behavior research methods, 39*(2), 175–191. 10.3758/bf03193146 17695343

[CR69] Stattin, H., & Kerr, M. (2000). Parental monitoring: A reinterpretation. *Child development, 71*(4), 1072–1085 10.1111/1467-8624.00210 11016567

[CR70] Lüdecke D (2024). *sjPlot: Data Visualization for Statistics in Social Science*. R package version 2.8.17. https://strengejacke.github.io/sjPlot/

[CR71] Frick, P. J., & Morris, A. S. (2004). Temperament and developmental pathways to conduct problems. *Journal of clinical child and adolescent psychology, 33*(1), 54–68. 10.1207/S15374424JCCP3301_6 15028541

